# Recognizing Kounis Syndrome: A Report of Type 2 Kounis Syndrome and a Brief Review of Management

**DOI:** 10.7759/cureus.19712

**Published:** 2021-11-18

**Authors:** Britney Clemen, Ifeanyi Nwosu, Nnamdi Chukwuka, Nikhil L Cordeiro, Emeka Ibeson, Amit Gulati, Sergey Ayzenberg, Benjamin Weindorf

**Affiliations:** 1 Internal Medicine, Maimonides Medical Center, New York City, USA; 2 Cardiology, Maimonides Medical Center, New York City, USA

**Keywords:** myocardial infarction, nut allergy, anginal chest pain, allergy and anaphylaxis, non-st segment elevation myocardial infarction (nstemi), coronary artery disease, allergic vasospastic angina, acute coronary syndrome, kounis syndrome

## Abstract

Kounis syndrome is an underdiagnosed medical condition and represents acute coronary syndrome in the setting of allergic reaction. With the increasing prevalence of allergic reactions, more cases of Kounis syndrome are being reported in the literature. Recognizing patients with acute coronary syndrome during an episode of anaphylaxis may be difficult due to symptom overlap; hence, a high index of suspicion must be maintained. This is vital as the management of Kounis syndrome requires meticulous use of medications as some pharmacological agents beneficial to acute coronary syndrome may be detrimental for the ongoing anaphylaxis and vice versa. We report a case of type 2 variant of Kounis syndrome following severe anaphylaxis to nuts to highlight the need for clinicians to suspect Kounis syndrome when managing patients with anaphylaxis and chest symptoms.

## Introduction

Anaphylaxis is a severe life-threatening form of type 1 hypersensitivity (allergic) reaction with multiple organ system involvements, a condition in which the cardiovascular system is both a victim and culprit of the disease. This is because the heart is the greatest source and target of chemical mediators of anaphylaxis [1,2]. Anaphylaxis is a medical emergency, and concurrent acute coronary syndrome (ACS) is a harbinger of disaster.

Kounis syndrome or allergic angina occurs when the response to the chemical mediators of anaphylaxis results in vasospastic angina, myocardial infarction, or coronary stent thrombosis [3].

Anaphylaxis typically has a dramatic presentation with alarming external physical features such that the physician may fail to identify a concurrent ACS. Symptom overlap between ACS and anaphylactoid reaction includes the sensation of chest tightness or discomfort from myocardial ischemia or bronchospasm, respectively. The diagnosis is often made retrospectively; thus, a high index of suspicion and early recognition is paramount to the successful management of Kounis syndrome.

Any substance can trigger anaphylaxis, although in most cases, foods (primarily nuts, cow milk, egg, and fish), insect stings, rubber latex, medications, and even radiocontrast are responsible. Sometimes no apparent trigger is identified, and this is known as idiopathic anaphylaxis [4].

Many studies have shown increasing lifetime prevalence of allergy and anaphylaxis [5-7] with an estimated lifetime risk of 0.02-2.0% [8]. As a result, more cases of Kounis syndrome are being reported in the literature. We report a case of Type 2 Kounis syndrome following food-induced anaphylaxis and emphasize the need for a high index of suspicion in at-risk patients presenting with anaphylaxis.

## Case presentation

A 67-year-old male with a past medical history of coronary artery disease (CAD) with two stents in the first diagonal branch of the left anterior descending artery six years before admission, hypertension, hyperlipidemia, glaucoma, and previous anaphylaxis to nuts presented to the emergency department (ED) with anaphylaxis after eating a nut-containing bar. Shortly after ingestion, he called emergency medical services (EMS) for hives and difficulty breathing. He was tachypneic, diaphoretic, and nauseous on arrival to the ED, tripoding with a hot-potato voice, and had oropharyngeal angioedema. Vital signs showed a temperature of 98.5 (F), heart rate of 73 beats per minute, blood pressure of 140/89 mmHg, respiratory rate of 19 cycles per minute, and oxygen saturation of 95% while on 6 liters of oxygen via nasal cannula.

After receiving epinephrine x3, methylprednisolone 125mg x2, and diphenhydramine the patient was still symptomatic. Epinephrine infusion was commenced at 1 microgram/minute for refractory anaphylaxis. ENT observed uvula edema without laryngeal edema on two separate fiberoptic exams. Given voice changes in the ED, the patient was admitted to the ventilator unit for monitoring of biphasic anaphylactic reaction. He was titrated off the epinephrine drip for lack of hemodynamic instability congruent with anaphylaxis.

**Figure 1:**
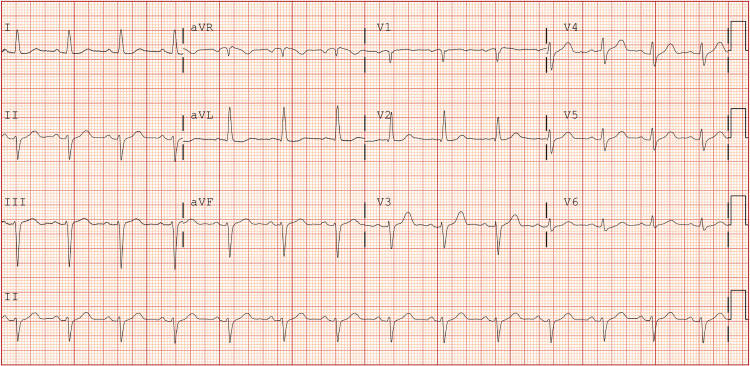
Electrocardiogram showing sinus rhythm, left axis deviation, and left anterior fascicular block

Initial laboratory investigations showed a troponin of 0.03ng/ml at 4.5 hours post-ingestion which increased to 2.98 and 3.08 at 18 and 23 hours post-ingestion, respectively. The patient initially denied chest pain; however, when informed of his cardiac enzyme elevation, he retrospectively endorsed intermittent, non-radiating, mid-sternal chest pressure that was 5/10 in intensity after receiving epinephrine, which decreased to 2/10 in intensity within 24 hours. Electrocardiogram (EKG) at 22 hours post-ingestion showed sinus rhythm, left anterior fascicular block, and no ST-segment changes (Figure 1).

He was started on aspirin, heparin infusion, and atorvastatin.

An echocardiogram showed a normal left ventricular (LV) systolic function with an ejection fraction of 56-60%. Additionally, LV segmental wall motion abnormalities, hypokinesis in the mid inferolateral segment were also found.

Chest X-ray did not show any abnormalities.

**Figure 2:**
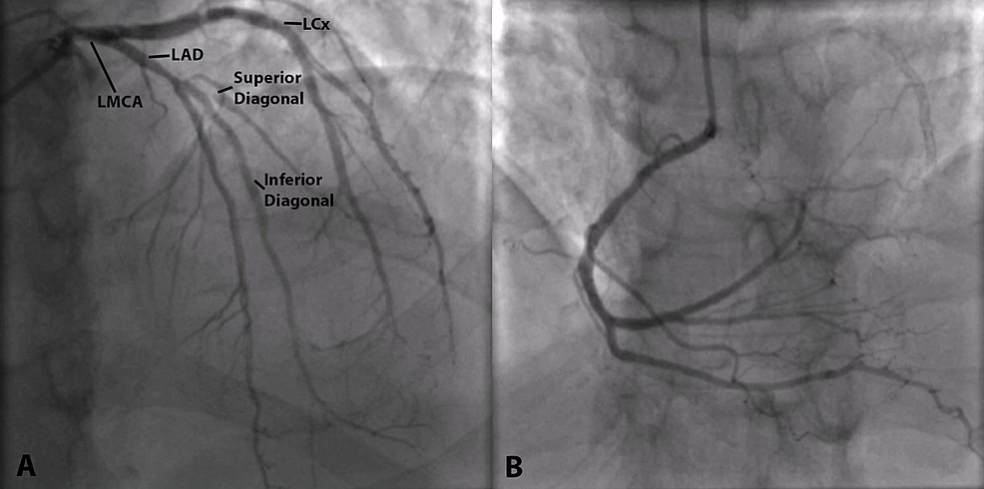
Coronary angiogram A. Anteroposterior caudal projection showing normal left main coronary artery (LMCA), normal left anterior descending artery (LAD), large diagonal branch with superior and inferior branches, patent stent extending from the mid-LAD to the inferior branch of the diagonal artery with 30% in-stent restenosis, jailed superior branch of the diagonal artery with 95% ostial lesion, left circumflex artery (LCx) with luminal irregularities. B. Left anterior oblique cranial projection showing normal right coronary artery (RCA) arising from the right sinus of Valsalva.

Elective coronary angiogram (Figure 2) was significant for single-vessel disease. The left main, left circumflex, and right coronary arteries were normal. Of note, a patent stent in the inferior branch of the diagonal artery with 30% in-stent restenosis and a jailed superior branch of the diagonal with 95% ostial lesion was also revealed.

Serial troponin monitoring showed a downward trend of 2.78 and 2.0ng/ml, at 30 and 39 hours post-ingestion, respectively. The patient's chest pain resolved, and he was discharged to follow up with the outpatient cardiology clinic.

## Discussion

Anaphylaxis follows immunoglobulin E (IgE)-mediated immune response to allergens following prior sensitization of B-cells resulting in specific IgE formation [8]. Antigen-dependent activation of tissue mast cells bound to specific IgE and blood basophils initiate the cascade of events in anaphylaxis [2,9]. Mast cells are present in various human tissues, including the skin and lungs, but are particularly abundant in the heart, more so in patients with ischemic heart disease and dilated cardiomyopathy [2]. Unlike other mast cells, cardiac mast cells can be activated by complement C3, C4, and radiocontrast [2]. Mast cell degranulation is accompanied by the release of chemical mediators of anaphylaxis, including histamine, tumor necrosis factor α (TNF-α), prostaglandin D2 (PGD2), platelet-activating factor (PAF), leukotrienes, chymase, renin, and other cytokines [2,10]. Many of these mediators have an opposing effect on various tissues. Histamine, PGD2, PAF, and leukotriene cause peripheral vasodilation, increased permeability, tissue edema, and bronchoconstriction characteristic of anaphylaxis [10]. Third spacing fluid shift and accompanying edema may result in significant hypotension, compromising coronary blood flow with myocardial ischemia [11].

Histamine causes vasoconstriction of coronary arteries mediated through the H1 receptors and PGD2, a coronary vasoconstrictor on the cardiopulmonary circulation [2,12]. This effect compromises coronary blood flow. Mediators of anaphylaxis also have the potential to increase thrombosis. Histamine, by inducing tissue factor expression in endothelial and vascular smooth muscle cells, activates factor X leading to thrombin formation. Furthermore, PAF can induce local platelet aggregations as well as cause atherosclerotic plaque instability and rupture [2]. Plaque rupture and thrombosis could result in impaired coronary blood flow and acute coronary syndrome.

The development of Kounis syndrome is a consequence of the above processes. Three types have been described as follows [13]. Type 1 describes patients without pre-existing coronary artery disease or risk factors who develop angina during an allergic reaction because of coronary artery spasm. Type 2 describes patients with a pre-existing, asymptomatic or symptomatic coronary artery disease in which an allergic reaction results in either coronary artery spasm or plaque erosion or rupture and myocardial infarction. Type 3 refers to allergic-induced coronary artery stent thrombosis (subtype a) and stent restenosis (subtype b) in patients with pre-existing coronary artery disease (CAD) and stents.

We believe our patient had allergic vasospastic angina in the setting of pre-existing coronary artery disease. Given the history of CAD and existing coronary stents, our patient is in the high-risk category for the development of ACS following anaphylaxis.

Effective management of Kounis syndrome entails treating anaphylaxis while ensuring adequate myocardial perfusion. Initial therapy should focus on aborting life-threatening anaphylaxis while bearing in mind that some of the medications may affect coronary blood flow. Treatment of anaphylaxis will minimize airway edema and obstruction as well as reduce interstitial tissue fluid losses resulting in improved tissue oxygenation and perfusion.

Epinephrine, although central to the management of anaphylaxis, may aggravate myocardial ischemia by worsening coronary artery spasm and potentially causing fatal arrhythmias [13,14]. However, there are no reports of fatal outcomes due to the use of intramuscular epinephrine in patients with Kounis syndrome. The use of H1 receptor blockers such as diphenhydramine and chlorpheniramine are not harmful in Kounis syndrome. Although hypotension and reduced tissue perfusion may result if administered too rapidly, such treatments reduce angioedema and pruritus associated with anaphylaxis [13]. The potent anti-inflammatory property of steroids makes them a frontline medication in reducing protracted anaphylaxis. There is a theoretical increased risk of a cardiac aneurysm and wall rupture in patients with myocardial infarction, but steroid use in Kounis syndrome is safe, effective, and beneficial to aborting anaphylaxis [11,13].

Once stabilized, treatment should focus on ACS following existing guidelines.

Antiplatelet therapy should be initiated in ACS following existing guidelines. Aspirin should be initiated in Kounis syndrome in patients without actual aspirin or non-steroidal anti-inflammatory drugs (NSAID) allergy. NSAIDs and aspirin act by inhibiting the cyclooxygenase pathway, reducing the synthesis of PGD2, which is of benefit as PGD2 causes coronary vasoconstriction; however, there is a potential to worsen anaphylaxis due to increased leukotriene production by the uninhibited lipoxygenase [13,15].

Special consideration must be given before the use of beta-blockers in Kounis syndrome. Generally, beta-blockers are incredibly beneficial in ACS management as it slows down the heart rate and reduces myocardial oxygen demand. Their use may be contraindicated in decompensated heart failure or high-degree heart block and patients with Kounis syndrome as they not only interfere with epinephrine but may worsen coronary artery spasm through an unopposed alpha-adrenergic receptor activity [11,16]. When necessary, glucagon may be administered in patients who are on long-term beta-blockers with anaphylaxis requiring epinephrine to minimize epinephrine resistance and risk of subsequent coronary artery spasm [13,16]. Morphine is another medication often used in ACS for analgesia as well as relief of anxiety which can worsen anaphylaxis by inducing degranulation of mast cells [15].

## Conclusions

ACS occurring during an anaphylactic reaction could result in a tragic outcome if not recognized on time. Although difficult to recognize due to symptom overlap between ACS and anaphylaxis, physicians must maintain a high index of suspicion when managing anaphylactic patients. This is primarily because the pharmacologic management of concurrent ACS and anaphylaxis is complicated by the fact that the individual mainstays of treatment for both ACS and anaphylaxis have exacerbating effects. Therefore, early recognition of Kounis syndrome could help direct the treatment of both ACS and anaphylaxis.
